# Genetic variants of the dUTPase-encoding gene *DUT* increase HR-HPV infection rate and cervical squamous cell carcinoma risk

**DOI:** 10.1038/s41598-018-36757-7

**Published:** 2019-01-24

**Authors:** Feng Ye, Hanzhi Wang, Jia Liu, Qi Cheng, Xiaojing Chen, Huaizeng Chen

**Affiliations:** 1 Women’s Reproductive Health Key Laboratory of Zhejiang Province, Women’s Hospital, School of Medicine, Zhejiang University, Hangzhou, Zhejiang, 310006 P. R. China; 2 Department of Obstetrics and Gynecology, Women’s Hospital, School of Medicine, Zhejiang University, Hangzhou, Zhejiang, 310006 P. R. China

## Abstract

Deoxyuridine 5′-triphosphate nucleotidohydrolase (dUTPase) is involved in the repair and prevention of uracil misincorporations into DNA. Maintenance of DNA integrity is critical for cancer prevention. Many studies have identified susceptibility loci and genetic variants in cervical cancer. The aim of this study was to explore the distribution frequency of six single nucleotide polymorphisms (SNPs) in the dUTPase-encoding gene *DUT* in a case-control study to identify the relationship between *DUT* genetic variants and cervical cancer susceptibility. Six *DUT* intronic SNPs (rs28381106, rs3784619, rs10851465, rs28381126, rs3784621 and rs11637235) were genotyped by mismatch amplification-PCR in 400 cervical squamous cell carcinomas (CSCCs), 400 precursor cervical intraepithelial neoplasia (CIN) III lesions and 1,200 normal controls. No correlations were found between four *DUT* SNPs (rs3784621, rs10851465, rs28381106 and rs28381126) and CIN III and CSCC risk. However, the homozygous GG allele of rs3784619 and TT allele of rs11637235 correlated significantly with increased risk of CIN III and CSCC (OR = 2.29, 2.05; OR = 3.15, 3.15, respectively). Individuals with the G allele or G carrier allele (AG + GG) at rs3784619 and with the T allele or T carrier allele (CT + TT) at rs11637235 were at higher risk for CIN III and CSCC (OR = 1.26, 1.30; OR = 1.41, 1.65, respectively). Similarly, in the human papillomavirus (HPV)-positive groups, we found that the homozygous GG alleles of rs3784619 and TT alleles of rs11637235 markedly increased the risk of CIN III and CSCC (OR = 2.44, 2.71; OR = 3.32, 4.04, respectively). When performing a stratified analysis of sexual and reproductive histories, we found that the GG genotype of rs3784619 had a particularly high level of enrichment in the group of patients with > one sexual partner in CIN III (P = 0.043) and CSCC (P = 0.007). Meanwhile, the TT genotype of rs11637235 was enriched for in the high risk HPV (HR-HPV)-positive cases of CIN III (P = 0.033) and CSCC (P = 0.022). Analysis of the haplotype between rs3784619 (A/G) and rs11637235 (C/T) revealed that the genotypes with AA-TT (OR = 2.59), AG-TT (OR = 2.29), GG-CC (OR = 2.72), GG-CT (OR = 3.01 (1.83–4.96)) were significantly associated with increased risk of CIN III. More notably, this risk was much greater for CSCC (AA-TT (OR = 3.62), AG-TT (OR = 5.08), GG-CC (OR = 5.28), and GG-CT (OR = 4.23). Additionally, most GG genotypes of rs3784619 were linkage GG-CT, while most TT genotypes of rs11637235 were linkage AA-TT. In conclusion, these findings suggested that the homozygous GG allele of rs3784619 and the TT allele of rs11637235 in the *DUT* gene significantly increased the risk of CIN III and CSCC. Most GG genotypes of rs3784619 and TT genotypes of rs11637235 were linkage GG-CT and AA-TT, respectively. The TT genotype of rs11637235 was enriched in the HR-HPV-positive cases. These two SNPs of the *DUT* gene can be early predictive biomarkers of CIN III and CSCC, and may be involved in HR HPV infection.

## Introduction

Uracil misincorporation occurs spontaneously at low levels as a result of cytosine deamination or misincorporation of deoxyuridine monophosphate (dUMP) during genomic DNA replication^1,2^. Under normal conditions, such lesions are rapidly repaired by the base excision repair mechanism initiated by uracil-DNA glycosylase enzymes^3,4^. The maintenance of DNA stability and integrity through DNA repair is critical in the prevention of cancer. Several factors are associated with an increased risk of cancer due to germline mutations in genes encoding DNA repair enzymes^5^. Five genes, *UNG*, *SMUG1*, *MBD4*, *TDG*, and *DUT*, are involved in the repair and prevention of uracil misincorporation into DNA, an anomaly that can lead to cancer-causing mutations. Little is known about the determinants of uracil misincorporation, including the effects of single nucleotide polymorphisms (SNPs) in the above-mentioned genes^6^. SNPs in DNA repair genes have been related to cancer risk as well^7,8^.

Cervical cancer is the third most common malignancy in women worldwide, and high risk human papillomavirus (HR-HPV) is the primary etiological agent^9^. In China, cervical cancer has become the most common female cancer (98.9 per 100,000) in addition to breast cancer. The cervical cancer mortality rate is 30.5 per 100,000, and the incidence rate is increasing^10^. However, although ~80% of women will acquire an HPV infection during their lifetime, only a small proportion of women will progress to develop invasive cancer^11^. Pedigree studies show that cervical cancer has a significant heritability factor, supporting a critical role of genetic susceptibility in cervical cancer etiology^12^.

Many studies, including two genome-wide association studies (GWAS), have identified susceptibility loci and genetic variants in cervical cancer^13–15^. However, these variants explain only some of the cervical cancer genetic susceptibility. Thus, additional susceptibility genetic loci need to be further explored.

The enzyme deoxyuridine 5′-triphosphate nucleotidohydrolase (dUTPase) is essential for the viability of cells in all organisms^16,17^. dUTPase is encoded by the *DUT* gene in humans. It catalyzes the hydrolysis of deoxyuridine triphosphate (dUTP) to dUMP and pyrophosphate, and thus removes dUTP from the DNA synthesis pathway. High levels of dUTP can result in uracil misincorporation^18^. Chanson *et al*. reported that the *DUT* SNP rs4775748 is associated with decreased uracil concentrations. This suggests that *DUT* SNPs may influence cancer risk because elevated uracil misincorporation may induce mutagenic lesions^6^.

Thus far there have been no reports investigating the association between SNPs in the *DUT* gene and tumor susceptibility. In this study, we selected six SNPs in the *DUT* gene with minor allele frequency values greater than 0.05, and explored their distribution frequency in a case-control study (400 cervical squamous cell carcinomas (CSCCs), 400 precursor cervical intraepithelial neoplasia (CIN) III lesions and 1,200 normal controls). Our goal was to identify the relationship between *DUT* genetic variants and cervical cancer susceptibility.

## Results

### Correlation of DUT SNP genotypes with CIN III and CSCC risk

Genotypic and allelic frequencies of *DUT* SNPS rs3784619, rs3784621, rs11637235, rs10851465, rs28381106 and rs28381126 are depicted in Table 1. Genotype distributions were in Hardy-Weinberg equilibrium.

**Table 1:** Correlation between DUT SNPs with the risk of CIN III and CSCCs.

DUT Genotype	Controls (1200 cases)		CIN III (400 cases)		adjusted OR* (95% CI)	*P*	CSCCs (400 cases)		adjusted OR* (95% CI)	*P*
	Cases	%	Cases	%			Cases	%		
**rs3784619**										
AA	645	53.8	192	48.0	1.00 (ref)		181	45.3	1.00 (ref)	
AG	461	38.4	144	36.0	1.05 (0.82–1.34)	0.702	136	34.0	1.05 (0.82–1.35)	0.698
GG	94	7.8	64	16.0	**2.29** (**1.60–3.27)**	**0.000**	83	20.8	**3.15** (**2.24–4.41)**	**0.000**
AG+GG	555	46.3	208	52.0	**1.26** (**1.00–1.58)**	**0.046**	219	54.8	**1.41** (**1.12–1.77)**	**0.003**
Allelic frequency										
Allele A	1751	73.0	528	66.0	1.00 (ref)		498	62.3	1.00 (ref)	
Allele G	649	27.0	272	34.0	**1.39** (**1.17–1.65)**	**0.000**	302	37.8	**1.64** (**1.38–1.94)**	**0.000**
**rs3784621**										
TT	379	31.6	129	32.3	1.00 (ref)		133	33.3	1.00 (ref)	
TC	577	48.1	183	45.8	0.93 (0.72–1.21)	0.594	169	42.3	0.84 (0.64–1.08)	0.176
CC	244	20.3	88	22.0	1.06 (0.77–1.45)	0.719	98	24.5	1.15 (0.84–1.56)	0.388
TC+CC	821	68.4	271	67.8	0.97 (0.76–1.24)	0.804	267	66.8	0.93 (0.73–1.18)	0.536
**Allelic frequency**										
Allele T	1335	55.6	441	55.1	1.00 (ref)		435	54.4	1.00 (ref)	
Allele C	1,065	44.4	359	44.9	1.02 (0.87–1.20)	0.805	365	45.6	1.05 (0.90–1.24)	0.538
**rs11637235**										
CC	561	46.8	161	40.3	1.00 (ref)		139	34.8	1.00 (ref)	
CT	493	41.1	153	38.3	1.08 (0.84–1.39)	0.543	147	36.8	1.20 (0.93–1.56)	0.165
TT	146	12.2	86	21.5	**2.05** (**1.49–2.82)**	**0.000**	114	28.5	**3.15** (**2.32–4.29)**	**0.000**
CT+TT	639	53.3	239	59.8	**1.30** (**1.04–1.64)**	**0.024**	261	65.3	**1.65** (**1.30–2.09)**	**0.000**
Allelic frequency										
Allele C	1615	67.3	475	59.4	1.00 (ref)		425	53.1	1.00 (ref)	
Allele T	785	32.7	325	40.6	**1.41** (**1.19–1.66)**	**0.000**	375	46.9	**1.82** (**1.54–2.14)**	**0.000**
**rs10851465**										
CC	403	33.6	127	31.8	1.00 (ref)		119	29.8	1.00 (ref)	
CT	577	48.1	185	46.3	1.02 (0.79–1.32)	0.896	192	48.0	1.13 (0.87–1.46)	0.371
TT	220	18.3	88	22.0	1.27 (0.92–1.74)	0.141	89	22.3	1.37 (1.00–1.89)	0.054
CT+TT	797	66.4	273	68.3	1.09 (0.85–1.39)	0.500	281	70.3	1.19 (0.93–1.53)	0.157
Allelic frequency										
Allele C	1383	57.6	439	54.9	1.00 (ref)		430	53.8	1.00 (ref)	
Allele T	1,017	42.4	361	45.1	1.12 (0.95–1.31)	0.174	370	46.3	1.17 (1.00–1.37)	0.056
**rs28381106**										
TT	1,014	84.5	351	87.8	1.00 (ref)		334	83.5	1.00 (ref)	
TG	184	15.3	48	12.0	0.75 (0.54–1.06)	0.103	66	16.5	1.09 (0.80–1.48)	0.587
GG	2	0.2	1	0.3	1.44 (0.13–15.80)	0.764	0	0.0	—	
TG+GG	186	15.5	49	12.3	0.76 (0.54–1.07)	0.113	66	16.5	1.08 (0.79–1.46)	0.634
Allelic frequency										
Allele T	2212	92.2	750	93.8	1.00 (ref)		734	91.8	1.00 (ref)	
Allele G	188	7.8	50	6.3	0.78 (0.57–1.08)	0.140	66	8.3	1.06 (0.79–1.42)	0.706
**rs28381126**										
GG	1,081	90.1	358	89.5	1.00 (ref)		349	87.3	1.00 (ref)	
GT	118	9.8	42	10.5	1.08 (0.74–1.56)	0.704	50	12.5	1.31 (0.92–1.87)	0.130
TT	1	0.1	0	0.0	—	—	1	0.3	3.10 (0.19–49.65)	0.424
GT+TT	119	9.9	42	10.5	1.07 (0.74–1.55)	0.737	51	12.8	1.33 (0.94–1.88)	0.112
Allelic frequency										
Allele G	2280	95.0	758	94.8	1.00 (ref)		748	93.5	1.00 (ref)	
Allele T	120	5.0	42	5.3	1.05 (0.73–1.51)	0.780	52	6.5	1.32 (0.94–1.85)	0.104

Underlined values show significant difference.

*The P-values are standardized by age, age at first intercourse, number of sexual partners, age at first full-term pregnancy and number of parities.

Our results indicate that four *DUT* SNPs, rs3784621, rs10851465, rs28381106 and rs28381126, were not correlated with CIN III and CSCC risk (Table 1).

The AA, AG, and GG frequencies of the *DUT* SNP rs3784619 in the controls were 53.8%, 38.4% and 7.8%, respectively, 48.0%, 36.0% and 16.0%, respectively, in the CIN III group, and 45.3%, 34.0% and 20.8%, respectively, in the CSCC group. These findings indicated that the homozygous GG allele of rs3784619 was associated with a significantly increased risk of the precursor lesion CIN III (OR = 2.29 (1.60–3.27), P = 0.000) and CSCC (OR = 3.15 (2.24–4.41), P = 0.000). We found that the frequency of the G allele at rs3784619 was significantly higher in CIN III (272/800, 34.0%) and CSCC (302/800, 37.8%) compared to normal controls (649/2400, 27.0%). The increased risk associated with the G allele and CIN III and CSCC were (OR = 1.39 (1.17–1.65), P = 0.000) and (OR = 1.64 (1.38–1.94), P = 0.000), respectively. Individuals with the G allele or a G carrier allele (AG + GG) at rs3784619 were at a higher risk for CIN III (OR = 1.26 (1.00–1.58), P = 0.046) or CSCC (OR = 1.41 (1.12–1.77), P = 0.003).

The frequencies of CC, CT, and TT for the rs11637235 SNP in the controls were 46.8%, 41.1% and 12.2%, respectively, 40.3%, 38.3% and 21.5%, respectively, in the CIN III group, and 34.8%, 36.8% and 28.5%, respectively, in the CSCC group. These findings indicated that the homozygous TT allele of the rs11637235 SNP was associated with an increased risk of CIN III (OR = 2.05 (1.49–2.82), P = 0.000) or CSCC (OR = 3.15(2.32–4.29), P = 0.000). The frequency of the T allele at the rs11637235 SNP was significantly higher in CIN III (325/800, 40.6%) and CSCC (375/800, 46.9%) groups compared to controls (785/2400, 32.7%). The increased risk of the T allele in association with CIN III and CSCC were 1.41 (1.19–1.66) and 1.82 (1.54–2.14), respectively. The T allele or the T carrier allele (CT + TT) at rs11637235 was associated with a higher risk for development of CIN III (OR = 1.30 (1.04–1.64), P = 0.024) and CSCC (OR = 1.65 (1.30–2.09), P = 0.000).

### Correlation of DUT SNP genotypes with HR-HPV-positive CIN III and CSCC risk

In the HR-HPV-positive group, *DUT* SNPs rs3784621, rs10851465, rs28381106 and rs28381126 were not correlated with CIN III or CSCC risk. However, the homozygous GG allele of the rs3784619 SNP was associated with an increased risk of CIN III (OR = 2.44 (1.32–4.49, P = 0.004) and CSCC (OR = 3.32 (1.73–6.38), P = 0.000). The increased risk of the G allele for CIN III and CSCC development were OR = 1.44 (1.09–1.90) and 1.73 (1.27–2.35), respectively (Table 2).

**Table 2:** Correlation between DUT SNPs with the risk of CIN III and CSCCs in HPV-positive cases.

DUT Genotype	Controls (191 cases)		CIN III (310 cases)		adjusted OR* (95% CI)	*P*	CSCCs (178 cases)		adjusted OR* (95% CI)	*P*
	cases	%	cases	%			cases	%		
**rs3784619**										
AA	101	52.9	145	46.8	1.00 (ref)		76	42.7	1.00 (ref)	
AG	74	38.7	109	35.2	1.03 (0.70–1.52)	0.897	62	34.8	1.11 (0.71–1.75)	0.640
GG	16	8.4	56	18.1	**2.44** (**1.32–4.49)**	**0.004**	40	22.5	**3.32** (**1.73–6.38)**	**0.000**
AG+GG	90	47.1	165	53.2	1.28 (0.89–1.83)	0.185	102	57.3	1.51 (1.00–2.27)	0.051
Allelic frequency										
Allele A	276	72.3	399	64.4	1.00 (ref)		214	60.1	1.00 (ref)	
Allele G	106	27.7	221	35.6	**1.44** (**1.09–1.90)**	**0.010**	142	39.9	**1.73** (**1.27–2.35)**	**0.001**
**rs3784621**										
TT	58	30.4	107	34.5	1.00 (ref)		62	34.8	1.00 (ref)	
TC	88	46.1	141	45.5	0.87 (0.57–1.32)	0.507	71	39.9	0.76 (0.47–1.21)	0.246
CC	45	23.6	62	20.0	0.75 (0.45–1.23)	0.252	45	25.3	0.94 (0.54–1.62)	0.811
TC+CC	133	69.6	203	65.5	0.83 (0.56–1.22)	0.337	116	65.2	0.82 (0.53–1.26)	0.361
Allelic frequency										
Allele T	204	53.4	355	57.3	1.00 (ref)		195	54.8	1.00 (ref)	
Allele C	178	46.6	265	42.7	0.86 (0.66–1.11)	0.233	161	45.2	0.95(0.71–1.26)	0.709
**rs11637235**										
CC	92	48.2	119	38.4	1.00 (ref)		56	31.5	1.00 (ref)	
CT	75	39.3	107	34.5	1.10 (0.74–1.65)	0.632	63	35.4	1.38 (0.86–2.21)	0.181
TT	24	12.6	84	27.1	**2.71** (**1.59–4.59)**	**0.000**	59	33.1	**4.04(2.26–7.21)**	**0.000**
CT+TT	99	51.8	191	61.6	**1.49** (**1.04–2.15)**	**0.032**	122	68.5	**2.03** (**1.32–3.10)**	**0.001**
Allelic frequency										
Allele C	259	67.8	345	55.6	1.00 (ref)		175	49.2	1.00(ref)	
Allele T	123	32.2	275	44.4	**1.68** (**1.29–2.19)**	**0.000**	181	50.8	**2.18** (**1.62–2.94)**	**0.000**
**rs10851465**										
CC	66	34.6	94	30.3	1.00 (ref)		51	28.7	1.00 (ref)	
CT	87	45.5	139	44.8	1.12 (0.74–1.70)	0.586	82	46.1	1.22 (0.76–1.96)	0.411
TT	38	19.9	77	24.8	1.42 (0.86–2.35)	0.167	45	25.3	1.53(0.87–2.70)	0.139
CT+TT	125	65.4	216	69.7	1.21 (0.83–1.78)	0.324	127	71.3	1.32 (0.85–2.04)	0.224
Allelic frequency										
Allele C	219	57.3	327	52.7	1.00 (ref)		184	51.7	1.00 (ref)	
Allele T	163	42.7	293	47.3	1.20 (0.93–1.56)	0.157	172	48.3	1.26 (0.94–1.68)	0.124
**rs28381106**										
TT	163	85.3	277	89.4	1.00 (ref)		151	84.8	1.00 (ref)	
TG	28	14.7	32	10.3	0.67 (0.39–1.16)	0.152	27	15.2	1.04 (0.59–1.85)	0.891
GG	0	0.0	1	0.3	—	—	0	0.0	—	—
TG+GG	28	14.7	33	10.6	—	—	27	15.2	—	—
Allelic frequency										
Allele T	354	92.7	586	94.5	1.00 (ref)		329	92.4	1.00 (ref)	
Allele G	28	7.3	34	5.5	0.73 (0.44–1.23)	0.240	27	7.6	1.04 (0.60–1.80)	0.895
**rs28381126**										
GG	169	88.5	268	86.5	1.00 (ref)		163	91.6	1.00 (ref)	
GT	22	11.5	42	13.5	1.20 (0.69–2.09)	0.509	15	8.4	0.71 (0.35–1.41)	0.325
TT	0	0.0	0	0.0	—	—	0	0.0	—	—
GT+TT	22	11.5	42	13.5	—	—	15	8.4	—	—
Allelic frequency										
Allele G	360	94.2	578	93.2	1.00 (ref)		341	95.8	1.00 (ref)	
Allele T	22	5.8	42	6.8	1.19 (0.70–2.03)	0.524	15	4.2	0.72 (0.37–1.41)	0.338

Underlined values show significant difference.

*The P-values are standardized by age, age at first intercourse, number of sexual partners, age at first full-term pregnancy and number of parities.

The homozygous TT allele in the rs11637235 SNP was also associated with an increased risk of CIN III (OR = 2.71 (1.59–4.59), P = 0.000) and CSCC (OR = 4.04 (2.26–7.21), P = 0.000). The increased risk of the T allele with CIN III and CSCC were OR = 1.68 (1.29–2.19) and 2.18 (1.62–2.94), respectively.

### Association between DUT rs3784619, rs11637235 polymorphism with the sexual behavior and reproductive history in CIN III or CSCCs

Cases were designated into two groups according to sexual behavior and reproductive history. A stratified analysis was then performed with the *DUT* rs3784619 (A/G) and rs11637235 (C/T) genotypes (Table 3). We found that there was higher enrichment of the rs3784619 GG genotype for CIN III (χ^2^ = 4.089, P = 0.043) and CSCC (χ^2^ = 7.228, P = 0.007) when the patient had more than one sexual partner.

**Table 3:** Association between DUT rs3784619 polymorphisms and the risk for CIN III and CSCCs stratified by the sexual, reproductive history.

High risk exposure	Controls						χ^2^	*P*	CIN III						χ^2^	*P*	CSCCs						χ^2^	*P*
	AA		AG		GG				AA		AG		GG				AA		AG		GG			
	N	%	N	%	N	%			N	%	N	%	N	%			N	%	N	%	N	%		
**Age**																								
≤40	318	52.8	226	37.5	58	9.6	1.308	0.253	121	46.9	97	37.6	40	15.5	0.115	0.734	70	43.8	56	35.0	34	21.3	0.199	0.656
>40	327	54.7	235	39.3	36	6.0			71	50.0	47	33.1	24	16.9			111	46.3	80	33.3	49	20.4		
**Number of sexual partners**																								
≤1	519	53.9	372	38.6	72	7.5	0.165	0.685	158	50.0	114	36.1	44	13.9	**4.089**	**0.043**	146	47.2	112	36.2	51	16.5	**7.228**	**0.007**
>1	126	53.2	89	37.6	22	9.3			34	40.5	30	35.7	20	23.8			35	38.5	24	26.4	32	35.2		
**Age at the first intercourse**																								
≤20	196	54.6	134	37.3	29	8.1	0.087	0.768	60	46.2	45	34.6	25	19.2	0.717	0.397	54	43.2	42	33.6	29	23.2	0.557	0.455
>20	449	53.4	327	38.9	65	7.7			132	48.9	99	36.7	39	14.4			127	46.2	94	34.2	54	19.6		
**Number of parities**																								
≤3	301	54.9	207	37.8	40	7.3	0.681	0.409	81	51.3	53	33.5	24	15.2	0.933	0.334	64	48.9	44	33.6	23	17.6	1.439	0.230
>3	344	52.8	254	39.0	54	8.3			111	45.9	91	37.6	40	16.5			117	43.5	92	34.2	60	22.3		
**Age at the first parity**																								
≤22	119	50.6	94	40.0	22	9.4	1.421	0.233	42	46.2	32	35.2	17	18.7	0.365	0.546	43	48.3	26	29.2	20	22.5	0.088	0.767
>22	526	54.5	367	38.0	72	7.5			150	48.5	112	36.2	47	15.2			138	44.4	110	35.4	63	20.3		
**HR-HPV infection status**																								
Positive	101	52.9	74	38.7	16	8.4	0.138	0.711	145	46.8	109	35.2	56	18.1	0.807	0.369	76	42.7	62	34.8	40	22.5	0.621	0.431
Negative	225	53.8	165	39.5	28	6.7			24	51.1	18	38.3	5	10.6			11	47.8	9	39.1	3	13.0		

Underlined values show significant difference.

Stratified analysis was applied by the Kruskale Wallis H. A P value less than 0.05 was considered significant.

Enrichment was only found for rs11637235 (C/T) (Table 4) when HR-HPV infection was positive in the CIN III (χ^2^ = 4.542, P = 0.033) and CSCC (χ^2^ = 5.226, P = 0.022) groups.

**Table 4:** Correlation between DUT rs11637235 polymorphisms with the risk of CIN III and CSCCs stratified analysis by the sexual behavior, reproductive history.

High risk exposure	Controls						χ^2^	*P*	CIN III						χ^2^	*P*	CSCCs						χ^2^	*P*
	CC		CT		TT				CC		CT		TT				CC		CT		TT			
	cases	%	cases	%	cases	%			cases	%	cases	%	cases	%			case	%	case	%	case	%		
**Age**																								
≤40	288	47.8	252	41.9	62	10.3	1.670	0.196	101	39.1	96	37.2	61	23.6	1.153	0.283	59	36.9	62	38.8	39	24.4	1.598	0.206
>40	273	45.7	241	40.3	84	14.0			60	42.3	57	40.1	25	17.6			80	33.3	85	35.4	75	31.3		
**Number of sexual partners**																								
≤1	444	46.1	401	41.6	118	12.3	0.662	0.416	123	38.9	118	37.3	75	23.7	2.925	0.087	103	33.3	118	38.2	88	28.5	0.458	0.498
>1	117	49.4	92	38.8	28	11.8			38	45.2	35	41.7	11	13.1			36	39.6	29	31.9	26	28.6		
**Age at the first intercourse**																								
≤20	157	43.7	161	44.8	41	11.4	0.943	0.332	55	42.3	44	33.8	31	23.8	0.003	0.956	42	33.6	52	41.6	31	24.8	0.156	0.693
>20	404	48.0	332	39.5	105	12.5			106	39.3	109	40.4	55	20.4			97	35.3	95	34.5	83	30.2		
**Number of parities**																								
≤3	254	46.4	232	42.3	62	11.3	0.004	0.950	71	44.9	58	36.7	29	18.4	2.779	0.096	43	32.8	47	35.9	41	31.3	0.675	0.411
å 3	307	47.1	261	40.0	84	12.9			90	37.2	95	39.3	57	23.6			96	35.7	100	37.2	73	27.1		
**Age at the first parity**																								
≤22	105	44.7	101	43.0	29	12.3	0.387	0.534	39	42.9	37	40.7	15	16.5	1.032	0.310	33	37.1	34	38.2	22	24.7	0.661	0.416
>22	456	47.3	392	40.6	117	12.1			122	39.5	116	37.5	71	23.0			106	34.1	113	36.3	92	29.6		
**HR-HPV infection status**																								
Positive	92	48.2	75	39.3	24	12.6	1.560	0.212	119	38.4	107	34.5	84	27.1	**4.542**	**0.033**	56	31.5	63	35.4	59	33.1	**5.226**	**0.022**
Negative	221	52.9	157	37.6	40	9.6			24	51.1	17	36.2	6	12.8			12	52.2	8	34.8	3	13.0		

Underlined values show significant difference.

### Linkage disequilibrium analysis between DUT rs3784619 and rs11637235 genotypes

We conducted a linkage disequilibrium analysis between the rs3784619 (A/G) and rs11637235 (C/T) genotypes based on the observation that these genotypes were associated with an increased risk of CIN III or cervical carcinoma. The frequencies of the nine genotypes are shown in Table 5. The GG (rs3784619)-TT (rs11637235) genotype was not detected in CIN III and CSCC, and was only detected in one case in a normal control. Compared to the AA (rs3784619)-CC (rs11637235) genotype, genotypes with AA-TT (OR = 2.59 (1.70–3.94), P = 0.000), AG-TT (OR = 2.29 (1.36–3.85), P = 0.002), GG-CC (OR = 2.72 (1.59–4.68), P = 0.000), and GG-CT (OR = 3.01 (1.83–4.96), P = 0.000) showed a significant correlation with increased risk for CIN III. More notably, this increased risk was higher for CSCC (AA-TT (OR = 3.62 (2.36–5.55), P = 0.000), AG-TT (OR = 5.08 (3.15–8.19), P = 0.000), GG-CC (OR = 5.28 (3.18–8.78), P = 0.000), and GG-CT (OR = 4.23 (2.56–6.99, P = 0.000)). This meant that the linkage pattern was at high risk for either the GG homozygote of rs3784619 (A/G) or the TT homozygote of rs11637235 (C/T).

**Table 5:** DUT haplotypes(rs3784619-rs11637235) in CIN III and CSCC cases.

DUT Genotype	All patients and controls									
	Controls		CIN III		adjusted OR* (95% CI)	*P*	CSCCs		adjusted OR* (95% CI)	*P*
	(1200 cases)		(400 cases)				(400 cases)			
	cases	%	cases	%			cases	%		
AA-CC	301	25.1	72	18.0	1.00(ref)		57	14.3	1.00(ref)	
AA-CT	252	21.0	63	15.8	1.05(0.72–1.52)	0.819	61	15.3	1.28(0.86–1.90)	0.227
AA-TT	92	7.7	57	14.3	**2.59(1.70–3.94)**	**0.000**	63	15.8	**3.62(2.36–5.55)**	**0.000**
AG-CC	217	18.1	61	15.3	1.17(0.80–1.72)	0.409	39	9.8	0.95(0.61–1.48)	0.817
AG-CT	191	15.9	54	13.5	1.18(0.79–1.76)	0.409	46	11.5	1.27(0.83–1.95)	0.272
AG-TT	53	4.4	29	7.3	**2.29(1.36–3.85)**	**0.002**	51	12.8	**5.08(3.15–8.19)**	**0.000**
GG-CC	43	3.6	28	7.0	**2.72(1.59–4.68)**	**0.000**	43	10.8	**5.28(3.18–8.78)**	**0.000**
GG-CT	50	4.2	36	9.0	**3.01(1.83–4.96)**	**0.000**	40	10.0	**4.23(2.56–6.99)**	**0.000**
GG-TT	1	0.1	0	0.0	—	—	0	0.0	—	—

Underlined values show significant difference. aHaplotypes were composed by two SNPs of DUT gene: rs3784619(A/G), rs11637235(C/T).

*The P-values are standardized by age, age at first intercourse, number of sexual partners, age at first full-term pregnancy and number of parities.

Additionally, most GG (rs3784619) genotypes were linkage GG-CT (36/64, 43/83) in the CIN III and CSCCs, while most TT (rs11637235) genotypes were linkage AA-TT(57/86, 63/109) in group CIN III or in group CSCCs. These results revealed that the distribution of GG (rs3784619) and TT (rs11637235) genotypes was mostly caused by linkage disequilibrium of the corresponding alleles. Thus, the specific linkage disequilibrium between rs3784619 (A/G) and rs11637235 (C/T) can be used as a predictor of CIN III and CSCC.

## Discussion

Mechanisms for the prevention of uracil misincorporation into DNA and the removal of misincorporated uracil from DNA are essential for maintaining genomic integrity. It has been shown that failure to remove misincorporated uracil can result in mutations or double stranded DNA breaks following DNA replication, ultimately leading to chromosomal abnormalities, both of which are hallmarks of cancer^2,19–21^. Mammalian cells have enzymes, such as dUTPases, that prevent the incorporation of uracil into DNA by dephosphorylating dUTP into dUMP. In humans, the only known UTPase is encoded by the *DUT* gene^2,22^. This gene encodes an essential enzyme for nucleotide metabolism. The encoded protein forms a ubiquitous, homotetrameric enzyme that hydrolyzes dUTP to dUMP and pyrophosphate. This process serves two cellular goals: providing a precursor (dUMP) for the synthesis of thymine nucleotides needed for DNA replication, and limiting intracellular dUTP levels. Elevated dUTP levels lead to increased incorporation of uracil into genomic DNA, which induces extensive excision repair mechanisms mediated by uracil glycosylase. High dUTP levels would lead to uracil misincorporation followed by excision repair, ultimately causing DNA fragmentation and cell death^18^. Furthermore, the redundant dUMP is used in a salvage pathway for synthesizing deoxythymidine triphosphate^23^.

Chanson *et al*. examined the relationship between 23 genetic variants in five uracil-processing genes and uracil concentrations in whole blood DNA in 431 participants of the Boston Puerto Rican Health Study^6^. They found that four SNPs in *DUT*, *UNG*, and *SMUG1* had a significant association with DNA uracil concentrations. The SNPs in *SMUG1* (rs2029166 and rs7296239) and *UNG* (rs34259) were associated with increased uracil concentrations, whereas the *DUT* SNP (rs4775748) was associated with decreased uracil concentrations. These results suggested that the four SNPs in *DUT*, *UNG*, and *SMUG1* may have a direct effect on uracil concentrations in normal cells, thereby affecting post-DNA replication uracil misincorporation rates, resulting mutations, and ultimately, cancer risk.

Anogenital HPV infections are the most common sexually-transmitted infections, with a prevalence of 70 million cases and an incidence of 14 million cases per year in the United States^24–26^. Fifteen HR-HPV subgenotypes can lead to cancer of the cervix, penis, vagina, vulva, and oropharynx^27^. HPV-16 and HPV-18 subtypes are the most prevalent HR-HPV subgenotypes in HPV-associated cancers, accounting for approximately 70% of cervical cancers, with the other HR-HPV subtypes (31, 33, 35, 39, 45, 51, 52, 56, 58, 59, 68, 73, and 82) account for the remaining cervical cancer cases^28^. Nearly all cervical cancers are HPV-associated, including CSCC, cervical adenocarcinomas, and other histological cervical tumors^27^. Most HR-HPV-related cervical infections are asymptomatic, and more than 90% of detected infections clear within about two years^29^.

Although several contributing factors in cervical cancer development have been identified, mainly intrinsic genetic factors and extrinsic factors such as HR-HPVs, genetic factors show great potential for use as susceptibility or prognostic indicators^30,31^. A large amount of epidemiological evidence supports that genetic variants are associated with cervical cancer risk^15^.

Broderick *et al*. detected novel germline sequence variations in *TDG*, *UNG* and *SMUG1* in colorectal cancer cases with familial aggregation, suggesting that these variants may play a role in disease susceptibility^32^. Other studies have described an association of a SNP in *MBD4* with increased risk of lung^33,34^ and esophageal cancer^35^.

Many literature studies report that there is a significant correlation between SNPs in DNA repair genes and the susceptibility to different cancers^7,36^. We previously found an association between SNPs of *XPD*, *XPG*, *PARP-1* repair genes and the susceptibility to CSCC and HR-HPV infections^37,38^.

To date, no studies have been performed to investigate the correlation between SNPs of the *DUT* gene and cancer susceptibility. The development of cervical carcinoma usually requires multiple stages, eventually developing from precursor CIN lesions to cervical malignant carcinoma. In the present study, we found that there was no associations between four SNPs of the *DUT* gene (rs3784621, rs10851465, rs28381106 and rs28381126) and increased risk of CIN III or CSCC. On the other hand, the homozygous GG allele of rs3784619 and TT allele of rs11637235 were associated with a higher risk of CIN III and CSCC. Individuals with the G allele or G carrier allele (AG + GG) at rs3784619 and with the T allele or T carrier allele (CT + TT) at rs11637235 were at higher risk for CIN III and CSCC. We also found that the homozygous GG allele of rs3784619 and the TT allele of rs11637235 had a higher risk of CIN III and CSCC. These results showed that the homozygous GG allele of rs3784619 and the homozygous TT allele of rs11637235 in the *DUT* gene may play a role in initiation and progression of precancerous lesions (CIN) and cervical carcinoma. The present study is the first to report an association between *DUT* rs3784619 and rs11637235 genetic variants and any solid tumor type.

SNP loci that affect the spatial structure and function of genes are typically located in the 5′ UTR promoter, coding region, or 3′ UTR region. Although the two variants we investigated are both located in the intron, which cannot affect amino acid designation, it is still possible that there can be linkage disequilibrium with other functional genetic variants and therefore these variants can serve as a genetic biomarker of susceptibility^39^. The *DUT* rs3784619 and rs11637235 genetic variants could also influence primary mRNA splicing and regulation, and therefore could affect dUTPase protein expression or result in an alternative spliceosome. Very little is known about the functional effects of uracil-processing gene SNPs. It is thought that these SNPs can lead to altered enzyme activity, contributing to increased uracil concentrations and therefore uracil misincorporation, and ultimately resulting in human diseases including malignant cancers^2^.

When performing stratified analyses of the sexual and reproductive histories of patients, we found that the GG genotype of rs3784619 had a particularly high level of enrichment in the group with > one sexual partner for CIN III and CSCC. Meanwhile, the TT genotype of rs11637235 was enriched in HR-HPV-positive cases of CIN III and CSCC. These data suggested that there may be a correlation between the GG genotype of rs3784619 with female sexual behavior. The TT genotype of rs11637235 may affect HR-HPV infection at early onset of disease.

The analysis of the haplotypes of rs3784619 (A/G) and rs11637235 (C/T) revealed that the genotypes with AA-TT, AG-TT, GG-CC, and GG-CT were significantly associated with increased risk of CIN III and CSCC, with the risk being much greater for CSCC. Additionally, most GG genotypes of rs3784619 were linkage GG-CT, while most TT genotypes of rs11637235 were linkage AA-TT. Our data showed that the rs3784619 (A/G) and rs11637235 (C/T) polymorphisms of the *DUT* gene correlated with increased risk of CIN III and CSCC. Haplotype analysis showed a significantly greater risk of cancer occurrence particularly for the genotype combinations of linked GG-CT and AA-TT. These results revealed that the distribution of GG (rs3784619) and TT (rs11637235) genotypes was mostly caused by linkage disequilibrium of the corresponding alleles. Therefore, the significantly-higher odds ratios suggested a synergistic effect of polymorphisms in the *DUT* gene, and could be an important factor affecting susceptibility to CIN III or CSCC.

In conclusion, these findings shed light on two polymorphisms (rs3784619 and rs11637235) in the *DUT* gene associated with a higher risk of CIN III and CSCC that could be used as biomarkers. Most GG genotypes of rs3784619 and TT genotypes of rs11637235 were linkage to GG-CT and AA-TT, respectively. The two investigated SNPs of the *DUT* gene could be used as early predictive biomarkers of CIN III and CSCC, and in addition, may play a role in HR-HPV infection.

## Methods

### Ethics statement

This research project was authorized by the Medical Ethical Committee of Women’s Hospital, School of Medicine, Zhejiang University (approval number 2004002). All patients provided written informed consent to participate in the study. The research methods protocol was carried out in accordance with approved guidelines and regulations.

### Study subject selection, sexual and reproductive histories, and HR-HPV infection status

Four-hundred CSCC patients, 400 CIN III patients, and 1,200 normal control volunteers were selected from Zhejiang Province, China. Pathological diagnoses were made by two pathologists under double blind conditions. Normal controls were healthy female volunteers attending the hospital for routine physical examinations between June 2004 and December 2008. Normal control volunteers without gynecological neoplasm, cytological findings, endometriosis, other solid cancers or immune disorders were included. Of the included patients, 201 CSCC patients, 357 CIN III patients and 609 normal control volunteers agreed to provide cervical brush samples for HR-HPV detection.

The number of individuals younger than/older than 40 years old were 602/598, 258/142 and 160/240 for the normal controls, CIN III and CSCC groups, respectively. In the CSCC group, there were more individuals older than 40 years old (χ^2^ = 12.431, P < 0.001) compared to the normal control group. The CIN III group had more individuals younger than 40 years old (χ^2^ = 24.793,P < 0.001) compared to the normal control group.

Statistically significant differences were only identified for the number of parities in the CIN III and CSCC groups (χ^2^ = 4.627, P = 0.031; χ^2^ = 20.49, P < 0.001). Statistically significant differences were not observed for sexual and reproductive histories, including age at first intercourse (≤20 years old, >20 years old), number of sexual partners (≤1 partner, >1 partner) and age at first birth (≤22 years old, >22 years old) among the carcinoma, CIN III and control groups In the normal controls, CIN III and CSCC groups, the infection rate of HR-HPV was 31.4%, 86.8% and 88.6%, respectively. The infection rate of HR-HPV in the CIN III and CSCC groups were higher than in the control group (χ^2^ = 277.107, P < 0.001; χ^2^ = 199.315, P < 0.001).

### SNPs Selection

We searched the SNP status of the *DUT* gene with the SNP library option using the website for the National Center for Biotechnology Information, U.S. National Library of Medicine (https://www.ncbi.nlm.nih.gov/snp/). We utilized the filter option (filters activated: snp, minor allele frequency from 0.05 to 0.5, by-1000 Genomes, by-cluster, by-frequency, by-2hit-2allele) to obtain six effective SNPs in the *DUT* gene (rs3784619 (A/G), rs3784621 (C/T), rs11637235 (C/T), rs10851465 (C/T), rs28381106 (G/T) and rs28381126 (G/T)). All of these six SNPs are located in the intron of the DUT gene.

### DNA Extraction and Genotyping

A genomic DNA extraction kit was used to extract whole genomic DNA from peripheral white blood cells according to the manufacturer’s instructions (Sangon, Shanghai, China). All genomic DNA was dissolved in distilled water and frozen until further use.

The six intronic SNPs of the *DUT* gene were detected by a modified polymerase chain -mismatch amplification (MA-PCR) reaction as described previously^37^. PCR forward and reverse primer sequences and product lengths are showed in Table S1. In brief, the PCR was performed in a 30 µL reaction mixture, containing 50 ng of genomic DNA, 5.0 pmol of each primer, 0.2 mM of each dNTP and 1.5 units of Taq DNA polymerase (TAKARA, Dalian, China). The PCR reaction was performed with the following conditions: initial denaturation at 94 C for 5 minutes; followed by 35 cycles at 94 C for 30 seconds, 55–58 C for 30 seconds for annealing, and 72 C for 45 seconds for elongation. A final step of 72 C for 10 minutes was performed. PCR products were electrophoresed on a 1.5% agarose gel and visualized. All samples were tested twice by two different technicians in a double-blind fashion, with the reproducibility of assays being consistent.

### HR-HPV infection determination

HR-HPV infection detection was determined by the Hybrid Capture II assay kit (Digene Inc., USA) using probe B, which contains a sets of RNA probes for HR-HPV type 16, 18, 31, 33, 35, 39, 45, 51, 52, 56, 58, 59, and 68. Cervical DNA sampling for HR-HPV testing was obtained from cervical brushings with the Digene Cervical Sampler.

### Statistical analysis

A binary logistic regression analysis was used to analyze the correlation between SNP genotypes of the *DUT* gene with risk of CIN III and CSCC. OR(odds ratio), CIs (95% confidence intervals) and P-values are indicated. The normal control group was used as a reference. Stratified analyses were conducted between the sexual behavior, reproductive history and genotype distribution of SNPs in the *DUT* gene with a Kruskal-Wallis H test. All statistical values are bilateral. Statistical significance was recognized when P value were less than or equal to 0.05. All analyses were performed using SPSS statistical software version 18.0 for Windows.

## Electronic supplementary material

Table S1
