# Supplementary material for: Genetic variants of the dUTPase-encoding gene DUT increase HR-HPV infection rate and cervical squamous cell carcinoma risk
Source: Sci Rep. 2019 Jan 24;9:513. doi: 10.1038/s41598-018-36757-7 (PMC6345750; doi:10.1038/s41598-018-36757-7)
Supplement: Supplementary file 1 — Table S1 [file 41598_2018_36757_MOESM1_ESM.docx]

**Title:** Genetic variants of the dUTPase-encoding gene *DUT* increase HR-HPV infection rate and cervical squamous cell carcinoma risk

**Authors:** Feng Ye^1^, Hanzhi Wang^1^, Jia Liu^2^, Qi Cheng^1^, Xiaojing Chen^1^, Huaizeng Chen^1,^*

**Table S1:** The forward, reverse primers and PCR product length

| **SNP No.** | **Forward primer** | | **Reverse primer** | **Product length** |
| --- | --- | --- | --- | --- |
| rs28381106**[G/T]** | For “**T**” | 5’-AACGAAGATGCATCTGCCAT-3’ | 5’-ATGGTTCTAAGAACTGGGCA-3’ | 339bp |
|  | For “**G**” | 5’-AACGAAGATGCATCTGCCAG-3’ |  |  |
| rs3784619**[A/G]** | For “**A**” | 5’-ATCTCCAGGGGCCGTTCAGA-3’ | 5’-TAAGCTTTACTGTGTGCCA-3’ | 224bp |
|  | For “**G**” | 5’-ATCTCCAGGGGCCGTTCAGG-3’ |  |  |
| rs10851465**[C/T]** | For “**C**” | 5’-AAAAATTCATGTAAAAGATC-3’ | 5’-AGTGATTAACTGACTTCAGG-3’ | 341bp |
|  | For “**T**” | 5’-AAAAATTCATGTAAAAGATT-3’ |  |  |
| rs28381126**[G/T]** | For “**G**” | 5’-CAGAATTCACATCTGGTACG-3’ | 5’-AAAGCCTTCTCTGCCTACAG-3’ | 220bp |
|  | For “**T**” | 5’-CAGAATTCACATCTGGTACT-3’ |  |  |
| rs3784621**[C/T]** | For “**C**” | 5’-TTTTTATTTATGACTCAAAC-3’ | 5’-TCATGTAGCTCTGAGATTTG-3’ | 335bp |
|  | For “**T**” | 5’-TTTTTATTTATGACTCAAAT-3’ |  |  |
| rs11637235**[C/T]** | For “**C**” | 5’-AACCTTTAAAACCCAAAAC-3’ | 5’-GGAATACTAGAGGTGTAAGA-3’ | 310bp |
|  | For “**T**” | 5’-AACCTTTAAAACCCAAAAT-3’ |  |  |
